# Microfluidic Adaptation of Density-Gradient Centrifugation for Isolation of Particles and Cells

**DOI:** 10.3390/bioengineering4030067

**Published:** 2017-08-02

**Authors:** Yuxi Sun, Palaniappan Sethu

**Affiliations:** 1 Department of Biomedical Engineering, University of Alabama at Birmingham, Birmingham, AL 35294, USA; suny01@uab.edu; 2 Division of Cardiovascular Disease, Department of Medicine, University of Alabama at Birmingham, Birmingham, AL 35294, USA

**Keywords:** cell separations, label-free cell separation, microfluidics, density-gradient centrifugation

## Abstract

Density-gradient centrifugation is a label-free approach that has been extensively used for cell separations. Though elegant, this process is time-consuming (>30 min), subjects cells to high levels of stress (>350 g) and relies on user skill to enable fractionation of cells that layer as a narrow band between the density-gradient medium and platelet-rich plasma. We hypothesized that microfluidic adaptation of this technique could transform this process into a rapid fractionation approach where samples are separated in a continuous fashion while being exposed to lower levels of stress (<100 g) for shorter durations of time (<3 min). To demonstrate proof-of-concept, we designed a microfluidic density-gradient centrifugation device and constructed a setup to introduce samples and medium like Ficoll in a continuous, pump-less fashion where cells and particles can be exposed to centrifugal force and separated via different outlets. Proof-of-concept studies using binary mixtures of low-density polystyrene beads (1.02 g/cm^3^) and high-density silicon dioxide beads (2.2 g/cm^3^) with Ficoll–Paque (1.06 g/cm^3^) show that separation is indeed feasible with >99% separation efficiency suggesting that this approach can be further adapted for separation of cells.

## 1. Introduction

Cells in the body are either organized as complex multi-cellular tissue or as heterogeneous mixtures in fluids such as blood. Separation of cells into different sub-populations is an essential step for various applications such as immune-phenotyping, tissue engineering and evaluation of systemic inflammation [1,2,3,4,5,6,7]. The focus of most cell separations approaches is the isolation of cells from blood as they provide important prognostic and diagnostic information [2,6,8]. Blood consists of plasma, erythrocytes, leukocytes and platelets. 

Leukocytes or white blood cells are responsible for maintenance of immune homeostasis and for protecting the body from injury and infections. Therefore, sampling leukocytes from a patient provides valuable information regarding the immediate immune and inflammatory status of the patient [3,8,9]. Cell separation approaches exploit differences in either physical properties or biochemical specificities of different cell types to accomplish separation of cells into different sub-populations. Commonly used techniques include erythrocyte or red blood cell lysis which relies on selective susceptibility of erythrocytes to lysis when suspended in an ammonium chloride buffer [10], density-gradient centrifugation which takes advantage of differences in mass density between mononuclear leukocytes and erythrocytes and granulocytes [11] and immuno-affinity separations which rely on antibody-cell surface antigen interactions to enable capture [12]. Leukocyte sub-populations provide superior diagnostic and prognostic information in comparison to total leukocytes. However, isolation of leukocytes into sub-populations requires the use of antibodies or methods like density-gradient centrifugation which can both lead to activation of leukocytes due to the leukocyte binding event [13] or due to high levels of stress [14] during the isolation process. Considering the fact that leukocytes are highly sensitive to isolation process-induced stress which can result in artificial leukocyte activation [14], it is important to develop antibody-free approaches which minimally stress cells during the isolation process.

Density-gradient centrifugation is one of the most commonly used separation methods for fractionation of leukocyte subpopulations from the perspective of efficiency, purity and cost. By exploiting density difference among leukocyte subpopulations and erythrocytes, less dense peripheral blood mononuclear cells (PBMCs) are enriched in a suspended buffy layer following >350 g centrifugation for 30 min. User skills are critical for extraction to ensure efficient fractionation via removal of the thin band of PBMCs layered in between the Ficoll–Paque layer and platelet-rich plasma. However, this approach imposes stress on the cells leading to activation of leukocytes [14].

Microfluidics provides a powerful platform for analysis of small biological samples via precise manipulation of the fluids. Several microfluidics-based approaches have been developed to isolate and analyze leukocyte populations. The most common microfluidic approaches for isolation of mononuclear leukocytes or peripheral blood mononuclear cells (PBMCs) have exploited size difference to achieve separation of target cells via either filtration or inertial focusing-based platforms [15,16,17]. However, these approaches have not found use in the clinical setting due to inherent limitations with these techniques to distinguish cells with small size difference. Microfluidic filtration approaches also have to deal with issues such as cell deformability and the tradeoffs between throughput and clogging of microfluidic filters [18]. Inertial focusing also relies on cell size differences to accomplish cell sorting but size difference among blood cells is not sufficient for PBMC isolation and may require significant sample dilution to work effectively [19]. Microfluidic magnetophoresis, dielectrophoresis and acoustophoretic devices have been developed and used either with or without antibodies but their throughput and separation efficiency have prevented widespread adoption in the clinic or research setting [17,20,21,22,23]. Therefore, while microfluidics provides new opportunities for cell separation with potential to minimize isolation process-induced activation of cells by minimizing stress and processing times, we have yet to see clinical adaptation of these techniques.

There have been several prior efforts that have utilized centrifugal force to drive fluids or achieve cellular separations using microfluidic approaches. However, their approaches do not accomplish high-fidelity miniaturization of conventional density-gradient centrifugation where red blood cells and polymorphonuclear cells (PMNs) are isolated from PBMCs in unique fractions. Al-Faqheri et al. present an excellent review summarizing centrifugal force-based microfluidic efforts for cell separations [24]. Other works of importance to the method discussed in this paper include a manuscript by Balter et al. who used centrifugal microfluidics to label and count leukocyte populations [25], a manuscript by Yu et al. where they use centrifugal forces to drive fluid flow and accomplish leukocyte capture on immuno-modified surfaces [26], Ramachandraiah et al. [27] developed a lab-on-a-DVD for labeling and counting of CD4^+^ cells, a centrifugally driven immunoassay where antibody-coated beads are transported via centrifugal forces and an ELISA-like readout is used to facilitate accurate dosing of VEGF [28], Schaff et al. [29] developed an immunoassay using centrifugal microfluidics for evaluation of biomarkers in blood, and another manuscript by Zhang et al. [30] where a centrifugal microfluidic platform was used to separate plasma from the blood cells and used to separate plasma and determine hematocrit. There are also few examples of density-gradient centrifugation using miniaturized platforms. Kinahan et al. [31] developed a spira mirabilis-inspired geometry for blood processing using density-gradient media. Later they developed a similar platform to fractionate mononuclear blood cells [32]. Rather than operate in a continuous mode, they developed a valving system to retain samples within a chamber during application of centrifugal force. Another paper by Moen et al. [33] describes a density-gradient process where total leukocytes are separated from red blood cells at high efficiency. Finally, Ukita et al. [34] developed a percoll gradient-based density-gradient centrifugation to separate beads of different densities. The technique presented in this paper is unique as it faithfully mimics conventional density-gradient centrifugation using Ficoll–Paque.

The major design challenge in miniaturizing conventional density-gradient centrifugation is to account for scaling effects that distinguish microscale fluid behavior from conventional macroscale effects. Fluid flow in microfluidic channels is primarily laminar. However, in low aspect ratio channels, at higher Reynolds number flows, when cells and particles are comparable to the size of the channel, the parabolic flow profile that develops in these channels results in a velocity gradient across the cross-section of the channel resulting in inertial lift forces that cause migration of cells and particles to the outer walls. The wall lift forces push back on the cells/particles and equilibrium is achieved, resulting in focusing close to the walls with larger particles closer to the wall and smaller particles further away. When rectangular channels are arranged in a curved/spiral fashion, secondary Deans forces also develop resulting in rotational effects on the flowing fluid. This results in a single focusing position close to the inner wall.

For our setup to work conceptually, we require the centrifugal forces to be much greater than the inertial lift forces rendering the focusing effects due to fluid flow in the channels irrelevant with minimal generation of secondary Deans forces, which can cause mixing of the two phases (sample and Ficoll-Paque) flowing side by side. This is achieved by achieving specific geometries for a given spinning speed to ensure that the Reynolds number, which dictates the magnitude of the inertial lift forces, and Deans number, which dictates the magnitude of forces that cause fluid rotation within the channels, are small enough to not affect the flow and separation within the microfluidic channels.

Re =ρuLμ

$$\mathrm{De}\;=\sqrt{\frac{d}{2r}\frac{\rho vd}{\mu }}$$

This paper details an approach that has great potential to be adapted for separation of PBMCs in the clinical setting. Conventional density-gradient centrifugation with Ficoll–Paque was miniaturized as a pump-free, continuous, label-free microfluidic system that, when mounted onto a custom built rotary platform, can enable separation of cells based on differences in density. While conceptually simple and straightforward, without minimization of inertial lift forces and Deans forces laminar flow of samples and Ficoll-Paque side-by-side will not be possible. This was accomplished via careful manipulation of channel dimensions, fluidic resistances, orientation of inlets and outlets and direction of rotation. To demonstrate successful proof of concept of this technique to separate cells/particles of different densities, we utilized low-density polystyrene beads (PS) (1.02 g/cm^3^) and high-density silicon dioxide (SD) beads (2.2 g/cm^3^) with Ficoll–Paque (1.06 g/cm^3^).

## 2. Materials and Methods 

### 2.1. Materials Microfluidic Device Fabrication

Microfluidic devices were fabricated using methods previously established in our laboratory [35]. Briefly, a 2D layout of the channel architecture was created using AutoCAD layout software (Autodesk, Inc., San Rafael, CA, USA) and printed using a high-resolution printer on a mylar sheet (Fineline Imaging, Colorado Springs, CO, USA). This photolithography mask was then used to define channel structures using a negative photoresist (SU-8 50, Microchem Corp, Westborough, MA, USA) on a silicon wafer. Using standard soft-lithography, the microfluidic devices were molded using (poly)dimethylsiloxane (PDMS) (Dow Corning, Midland, MI, USA) and bonded to either a silicon or glass wafer. Access holes for the 2 inlets and 2 outlets were punched using a 22-gauge blunt syringe needle and tubing was press-fitted to introduce and remove fluids. Two holes were also punched close to the center of the device to hold 2 mL Nalgene Cryogenic Vials (Thermo Scientific, Waltham, MA, USA) reservoirs. The caps of reservoirs were punched holes by 22-gauge needle for delivering samples contained within the reservoirs into the main channel via the connecting tubing.

### 2.2. Centrifugation System

**Figure 1:**
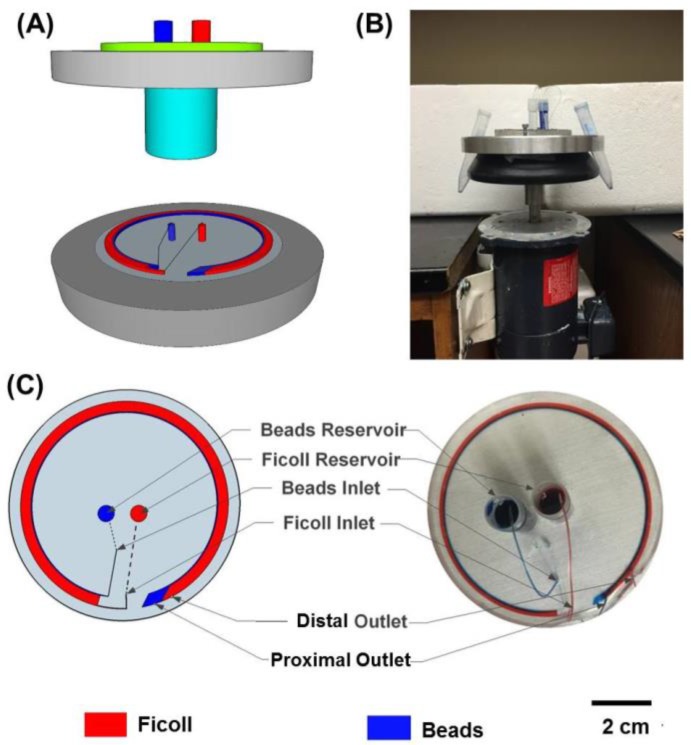
Setup for microfluidic density-gradient centrifugation. (**A**) Simplified schematic of the device; (**B**) Picture of the centrifugation system consisting of the custom-designed rotary platform mounted onto the motor to hold the microfluidic device; (**C**) Schematic and actual image of layering of colored 1X PBS streams within the device.

A system was designed and fabricated to enable microfluidic density-gradient centrifugation. The system consists of a variable-speed DC motor (AO Smith, Pitt City, OH, USA) and a custom-designed rotary platform that can be mounted on the motor to hold the microfluidic device (Figure 1). The rotary platform has a machined slot to hold the silicon wafer in place during spinning and two slots at diametrically opposite locations to collect the samples from the two outlets. The system spins clockwise with maximum speed of 1725 rpm.

### 2.3. Particles and Fluids

To demonstrate proof-of-concept, we used 2 different particles with different densities and a Ficoll–Paque solution with an intermediate density. Specifically, we used low-density fluorescently labeled polystyrene beads (1.02 g/cm^3^) (Thermo Fisher Scientific, Waltham, MA, USA) and high-density silicon dioxide beads (2.2 g/cm^3^) (Thermo Fisher Scientific, Waltham, MA, USA) with Ficoll–Paque (1.06 g/cm^3^) (GE Healthcare, Uppsala, Sweden). The particles were suspended as a 2% solution in 1X phosphate buffered saline (PBS) with 1% bovine serum albumin (BSA) to prevent aggregation. The solutions were vigorously shaken prior to use, loaded in beads reservoir along with Ficoll–Paque and accelerated rapidly for 5 min and then gradually decelerated until the system came to rest.

### 2.4. Flow Characterization

1X (PBS) (Thermo Fisher Scientific, Waltham, WA, USA) and Ficoll–Paque Plus (GE Healthcare, Uppsala, Sweden) were loaded in each reservoir and the centrifugation system was operated between 400-1600 rpm for 5 min (*n* = 3). After spinning stops, the inlet reservoirs and outlet collection tubes were removed and the quantity of liquids in each was measured to estimate the collective and relative flow rates. 

### 2.5. Evaluation of Samples

Evaluation of samples within the channels and collected in the reservoirs were imaged using bright-field and fluorescence microscopy (Nikon TE 2000, Nikon Instruments, Melville, NY, USA). For evaluation of beads within the channels, PDMS devices bonded to glass were directly imaged at different locations. Samples collected in the reservoirs were analyzed using a hemocytometer. Fluorescently labeled polystyrene beads were distinguished from silicon dioxide beads via fluorescence imaging.

## 3. Results

### 3.1. Device Dimensions for Optimal Laminar Flow and Layering

Various designs were evaluated for establishment of optimal laminar flow and layering of the sample stream over the Ficoll–Paque stream. The design that produced the optimal results without inducing mixing due to inertial forces and Deans forces was 3 mm wide with a pitch of 45 mm (Figure 1). The total channel length was 20 cm and the channel ran along the circumference of the silicon/glass wafer with room provided for the inlets and outlets. The width of the channels was 3 mm and the height was 50 µm, resulting in an aspect ratio of 60:1 (w:h). To avoid secondary forces that typically develop in microfluidic channels, we found that channel heights greater than 100 µm result in inertial forces that cause particle migration towards the side walls and Deans flow resulting fluid rotation within the channels, which disrupts the laminar flow within the channels. Therefore, a channel height of 50 µm and a channel width of 3 mm were selected to avoid fluid rotation and inertial particle migration within the channels.

### 3.2. Characterization of Total and Relative Flow Rates

**Figure 2:**
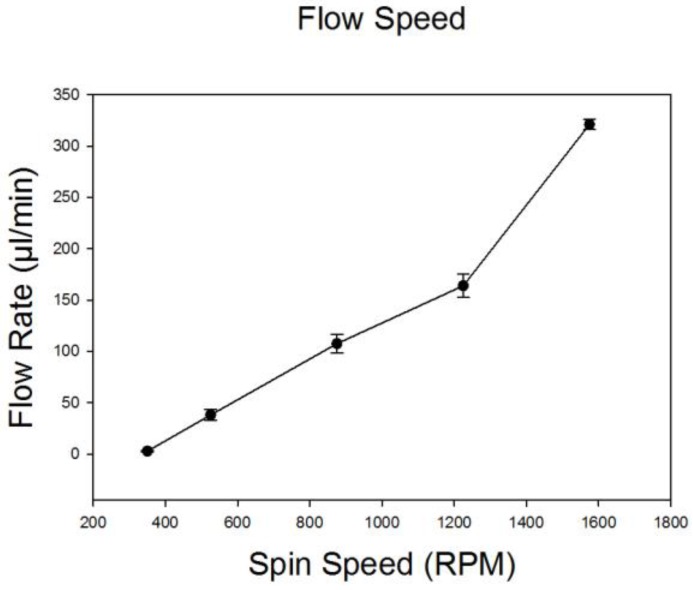
Characterization of fluid flow rates (1X PBS and Ficoll–Paque) at 400, 550, 900, 1250 and 1600 rpm (*n* = 3). The ratio of PBS to Ficoll was maintained constant at 1:4 by adjusting the resistances of the tubing.

To estimate the total and relative flow rates of particle samples and Ficoll–Paque, we measured the quantity of liquid in the inlet reservoirs and in the outlet collection tubes following spinning at different speeds ranging from 400 to 1600 rpm (Figure 2). The ratio of sample to Ficoll–Paque was maintained at 1:4 via adjustment of fluidic resistances leading into the main flow channel. This ratio remained relatively constant regardless of the spin speed and was ideal for particle separations. The total flow rate ranged from 50 mL/min at 550 rpm to a maximum flow rate of 330 mL/min at 1600 rpm. These results were consistent (*n* = 3) and the standard deviations negligible.

### 3.3. PS Bead Separation

**Figure 3:**
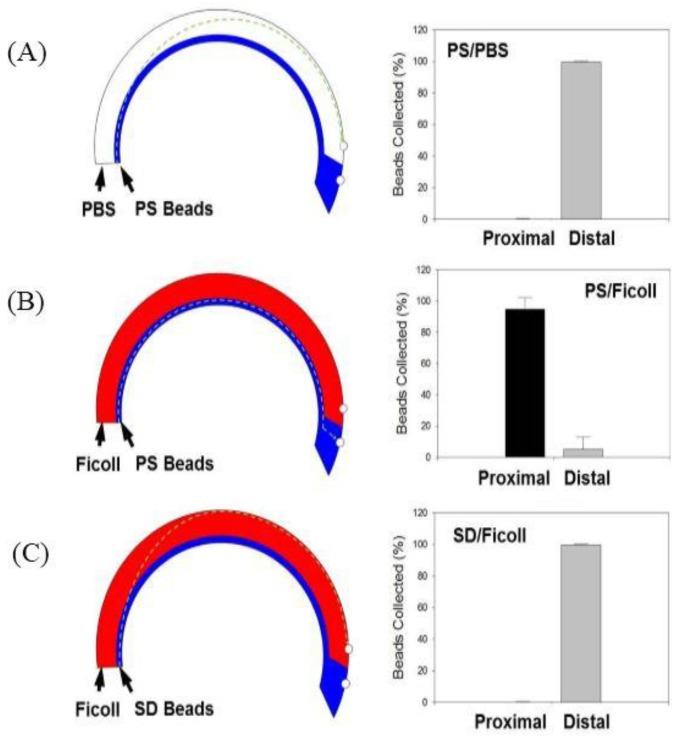
Proof-of-Concept Studies were established by flowing either polystyrene (PS) or silicon dioxide (SD) beads solutions with either 1X PBS or Ficoll to determine migration behavior and isolation via either the proximal or distal outlets. On the left is the schematic with hypothesized path of travel through the microfluidic channel at a speed of 875 rpm (40 g), and on the right is a plot with % of beads collected at each outlet for the following conditions: (**A**) PS Beads and 1X PBS; (**B**) PS Beads and Ficoll and (**C**) SD Beads and Ficoll (*n* = 5).

To demonstrate initial proof-of-concept, a 2% solution of PS beads (1.02 g/cm^3^) suspended in a 1X PBS solution was flowed into the system and layered over either 1X PBS (1.00 g/cm^3^) or Ficoll–Paque (1.06 g/cm^3^) and the device was spun at a speed of 875 rpm, which generates centrifugal force of ~40 g. As expected when layered over 1X PBS, the centrifugal force pushes the PS beads through the lower-density 1X PBS resulting in >99% of PS beads being collected via the distal outlet (Figure 3A). However, when the PS beads were layered over the higher-density Ficoll–Paque, the PS beads remain at the interface of the Ficoll–Paque layer unable to transit through the higher-density medium resulting in >98% of PS beads fractionated via the proximal outlet (Figure 3B).

3.4 SD Bead Separation

SD beads with a density of 2.2 g/cm^3^ are heavier than Ficoll–Paque and we sought to determine if we could isolate SD beads via the distal outlet when the device was spun at a speed of 875 rpm which generates centrifugal force of ~40 g. Results confirm that the heavier SD particles do indeed transit through the Ficoll–Paque and >99% of the beads can be fractionated via the distal outlet (Figure 3C). Results are represented as means ± SD (*n* = 5).

### 3.5. Separation of PS–SD Bead Mixture

**Figure 4:**
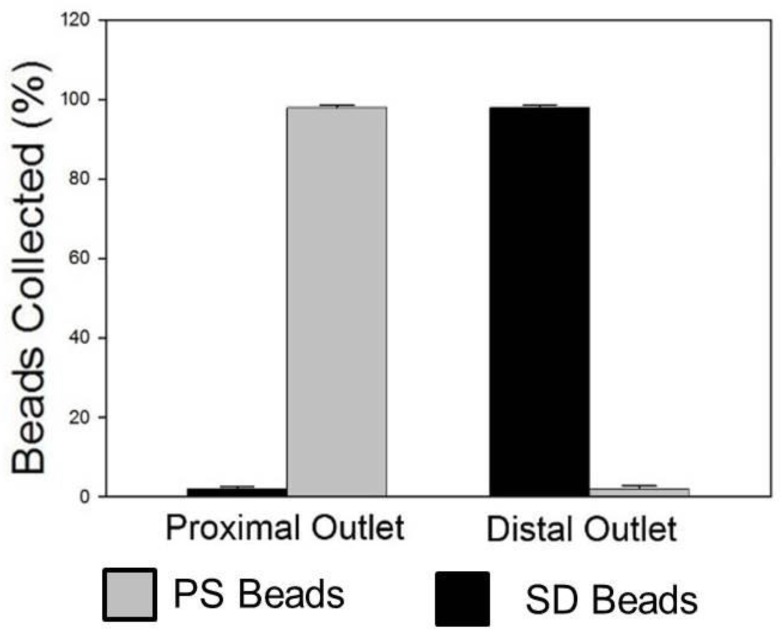
Quantitative assessment of beads collected via the proximal and distal outlets using a hemocytometer under bright-field imaging and fluorescence microscopy. Results show that >99% of PS beads were obtained via the proximal outlet, whereas >99% of SD beads were obtained via the distal outlet (*n* = 5).

**Figure 5:**
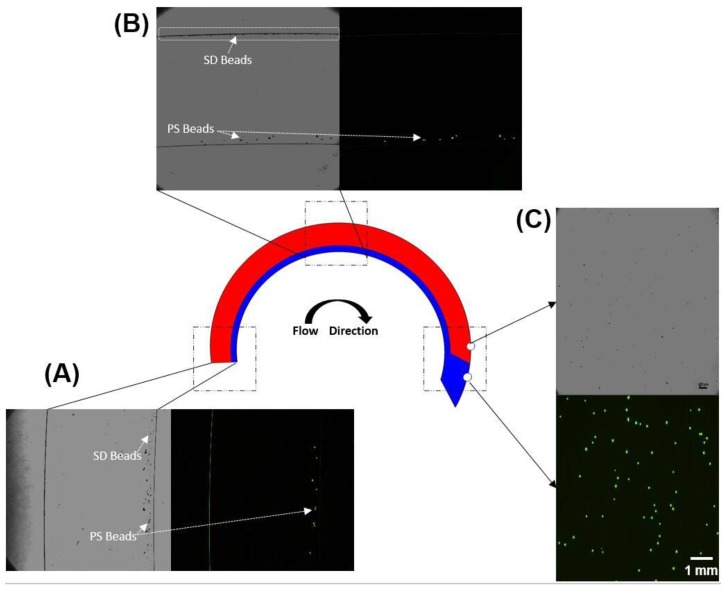
Images of PS (fluorescently labeled) and SD (unlabeled) beads at different locations during the microfluidic density-gradient centrifugation process. PS beads are visible in both the bright-field and fluorescence images, whereas the SD beads are only visible in the brightfield images. (**A**) At the inlet, both PS and SD beads are closer to the inner wall; (**B**) as the beads transit through the device, centrifugal force moves the heavier SD beads through the Ficoll and the SD beads can be seen close to the outer wall (highlighted region) whereas the PS beads are unable to migrate into the Ficoll and (**C**) Collected samples at the proximal and distal outlets confirm separation of PS and SD beads via the proximal and distal outlets respectively.

Finally, the ability of this device to achieve separation of particles of low- and high-density particles with a medium of intermediate density was accomplished. A 1X PBS solution containing a 4% solution of equal amounts of PS and SD beads was layered over Ficoll–Paque and the device was spun at 875 rpm (40 g). Results confirm that high-efficiency separation of PS and SD beads can indeed be accomplished using this approach with >99% PS beads collected via the proximal outlet and >99% of SD beads fractionated via the distal outlet (Figure 4). Results are represented as means ± SD (*n* = 5). Bright-field and fluorescence images of PS and SD beads at different locations (inlet, intermediate location within the channel and outlet) during this process are shown in Figure 5.

## 4. Discussion

Density-gradient centrifugation is an elegant technique that exploits differences in cell mass densities to achieve separation of PBMCs from erythrocytes and polymorphonuclear cells (PMNs). While this technique has been extensively used for over 50 years, shortcomings associated with high levels of stress imposed on cells, extended processing times and need for skilled technicians to cleanly isolate the fractionated samples have not been addressed. Microfluidics systems have great potential to miniaturize conventional macroscale separation approaches where samples confined in micrometer sized channels can be manipulated to enable faster, more precise and highly effective separations. To overcome the high levels of stress on cellular samples and minimize separation time, we sought to develop a microfluidic adaptation of conventional density-gradient separation process focused on minimizing duration and magnitude of centrifugation-induced stress on cells.

To accomplish this, we designed a system that could house a microfluidic device that was bonded to a 4’’ silicon wafer and subject it to rotary motion to induce centrifugal force for cell and particle separation. The microfluidic device itself consists of a channel where cell/particle samples can be layered as a laminar stream over medium like Ficoll–Paque. Within microfluidic devices, low Reynolds number flows ensure that viscous forces are dominant and laminar flow is achieved. In order to minimize inertial effects and potential Deans forces that can induce rotational mixing of the sample stream with the Ficoll–Paque, the width of the channel (3 mm) was significantly larger than the height of the channels (50 μm) resulting in an aspect ratio of 60:1 (w:h) and ensuring large interfacial contact area between the fluids and the device. This ensured that introduction of two streams of fluids with different viscosity and density can be maintained as laminar streams and the layering is maintained during rotational motion of the device. It is also critical that the samples flow direction is in the same direction of the rotary motion to avoid disruption of the layering process. Further, it is important to position and orient the inlet reservoirs and inlets correctly to ensure proper flow of samples and Ficoll–Paque into the device. In our pump-less system, centrifugal force was used to induce fluid flow by ensuring that the outlets were placed further from the center of the wafer than the inlets. The ratio of fluids was adjusted by controlling the fluidic resistances (tubing and inlet channel length and diameter). To achieve proper fractionation, the fluidic resistance of the two outlets was adjusted to ensure separation of low- and high-density particles. Finally, to avoid trapping and retention of the high-density particles within the channels, the spin speed (magnitude of centrifugal force) needs to be controlled to ensure that the high-density particles travel into the Ficoll–Paque layer but do not travel all the way to the outer wall.

Prior to optimizing this system for separation of cells, we sought to demonstrate proof-of-concept using particles. Polystyrene (PS) particles have a lower mass density than Ficoll–Paque, whereas silicon dioxide (SD) particles have a higher mass density and provide ideal particles for feasibility demonstrations. Initially, to confirm that beads introduced within the system experienced centrifugal force and moved across the channel we tested PS beads in solution with PBS. Our results confirm that higher-density PS particles move through the lower density PBS and are collected via the distal outlet. When the same PS beads were layered over Ficoll–Paque, the higher-density Ficoll–Paque retarded the motion of the PS beads and the PS beads were collected via the proximal outlet. When SD beads were layered over Ficoll–Paque, the higher-density SD beads easily transited through the Ficoll–Paque and were collected via the distal outlet. Finally, when a binary mixture of PS and SD beads were layered over Ficoll–Paque, the low-density PS beads remained at the interface of the Ficoll–Paque layer and were collected via the proximal outlet, whereas the high-density SD beads transited through the Ficoll–Paque and were collected at the distal outlet. The separation efficiencies for all separations were >99% confirming that the conventional Density-Gradient Centrifugation can be effectively miniaturized.

We believe that this technique is directly translatable to separation of blood cells. We utilized a maximum spinning speed of 875 rpm which translates to a residence time of 16 seconds within the device. This speed was sufficient to generate enough centrifugal force to move SD beads (2.2 g/cm^3^) which have a significantly higher density than Ficoll–Paque (1.07 g/cm^3^) close to the outer wall. Increased spinning speeds resulted in pinching of SD beads against the walls due to higher centrifugal force, which prevents collection of SD beads out of the channels. For blood cells, we anticipate that based on the smaller density difference between red blood cells (1.08 g/cm^3^), granulocytes (1.077 g/cm^3^) and Ficoll–Paque, a higher spin speed (~2500 rpm) and longer residence time (42s) will be necessary to accomplish depletion of red blood cells and granulocytes. These calculations were made using a modified Stokes settling velocity equation.

## 5. Conclusions

In summary, we demonstrate successful microfluidic adaptation of conventional density-gradient centrifugation. Proof-of-concept studies demonstrate high-efficiency separation of low-density PS beads from high-density SD beads when separated using a medium like Ficoll–Paque. These results suggest that this approach can potentially be adapted for separation of PBMCs from whole blood.
